# Assessment of the Inter-Batch Variability of Microstructure Parameters in Topical Semisolids and Impact on the Demonstration of Equivalence

**DOI:** 10.3390/pharmaceutics11100503

**Published:** 2019-10-01

**Authors:** Víctor Mangas-Sanjuán, María Pleguezuelos-Villa, Matilde Merino-Sanjuán, Mª Jesús Hernández, Amparo Nácher, Alfredo García-Arieta, Daniel Peris, Irene Hidalgo, Lluís Soler, Marta Sallan, Virginia Merino

**Affiliations:** 1 Departamento de Farmacia y Tecnología Farmacéutica y Parasitología, Facultad de Farmacia, Universitat de València, Av. Vicente Andrés Estellés s/n, Burjassot, 46100 Valencia, Spainmaplevi@alumni.uv.es (M.P.-V.); matilde.merino@uv.es (M.M.-S.); amparo.nacher@uv.es (A.N.); 2 Instituto Interuniversitario de Investigación de Reconocimiento Molecular y Desarrollo Tecnológico (IDM), Universitat Politècnica de València, Universitat de València, 46100 Valencia, Spain; 3 Departament de Fisica de la Terra i Termodinàmica, Universitat de València, Vicente Andrés Estelles s/n. Burjassot, 46100 Valencia, Spain; M.Jesus.Hernandez@uv.es; 4 División de Farmacología y Evaluación Clínica, Departamento de Medicamentos de Uso Humano, Agencia Española de Medicamentos y Productos Sanitarios, Calle Campezo 1, Ed 8, 28022 Madrid, Spain; agarciaarieta@gmail.com; 5 Pharmacokinetics and Clinical Affairs Department, Strategy and Development Area, Kern Pharma S.L., Calle Venus 72, Terrassa, 08228 Barcelona, Spain; danielperis@outlook.es (D.P.); ihidalgom@kernpharma.com (I.H.); 6 Formulation and Late Scale Development Department, Strategy and Development Area. Kern Pharma S.L., Calle Venus 72, Terrassa, 08228 Barcelona, Spain; lsoler@kernpharma.com (L.S.); msallan@kernpharma.com (M.S.)

**Keywords:** microstructure, rheology, equivalence, generic semisolid formulation, topical drug, inter-batch variability

## Abstract

Demonstration of similar microstructure is essential for demonstrating the equivalence of generic topical products since the microstructure of semisolids may affect the drug release. The objective of this study was to compare the microstructure-defining physical parameters of different batches of a reference ointment containing calcipotriol and betamethasone (Daivobet 50 µg/0.5 mg/g) in order to define the acceptance range that allows concluding equivalence between these batches. Being batches of the same reference product, they are expected to be clinically equivalent and possess similar microstructure. The 90% confidence intervals for the test/reference ratio of these physical parameters were calculated with parametric and non-parametric approaches. Both methods conclude that equivalent microstructure between batches cannot be demonstrated with a reasonable sample size when the acceptance range was set at ±10%, since several physical parameters exhibit inter-batch variability >10%. An acceptance range of ±10% is therefore too strict to conclude equivalence in the microstructure of semisolid dosage forms, given the inter-batch variability observed between batches of the reference product. A wider fixed acceptance range or an acceptance range widened based on the inter-batch variability of the reference product would be advisable.

## 1. Introduction

The arrangement of the matter in the structure of a semisolid product, also known as microstructure [1], is determined by the components employed in its formulation (e.g., polymorphism, size and shape of the dispersed particles of the active substance, grade and rheology of the excipients, interfacial tension between phases, partition coefficient of the active substance between the different phases) and the manufacturing process. This microstructure and the excipient composition do not only affect the organoleptic properties of the product, but also the drug permeation through the skin [1,2,3].

The European Medicines Agency (EMA) has released for consultation a draft guideline on the quality and equivalence of topical products [4]. The characterization of topical semisolids includes the investigation of their physical properties, which are defined by their microstructure, influence bioavailability and usability of the product and are indicative of stability and consistency of the manufacturing process. This draft guideline assumes that if the physical properties of two products with similar qualitative and quantitative composition are sufficiently similar, the microstructure of the formulation can also be considered similar.

According to this draft guideline, the demonstration of therapeutic equivalence of two products requires comparison of the qualitative (Q1) and quantitative (Q2) composition of the formulations, as well as a comparison of these physical properties (Q3). This comparison should be conducted in at least three batches of the reference product and three batches of the test product with at least 12 replicates per batch.

The microstructure characterization includes rheology, type of emulsion and physical state of drug in semisolid system (polymorphic form, solubilized drug vs. dispersed solid drug, particle size of drug particles) [1]. The draft guideline specifies that the non-Newtonian rheological behavior has to be characterized by investigating the complete flow curve (viscosity or shear stress versus shear rate) and the linear viscoelastic response (creep-recovery tests or oscillatory measurements at different frequencies). This allows calculation of parameters of viscosities at specified shear rates, yield stress values, thixotropic relative area (S_R_), elastic and viscous moduli (G’ and G″), or loss tangent (tan *δ*). To conclude equivalence of these microstructure characteristics, the 90% confidence interval (CI) of the difference between means of both formulations should not be more than 10% from the reference product mean, assuming normal distribution of data. According to this draft guideline, equivalence has to be concluded for all rheological parameters that define the microstructure of the product. Neither the parameters that define the microstructure of semisolid pharmaceuticals, nor the acceptance limits that ensure equivalence in those parameters have so far been defined in the literature. Moreover, since all the rheological parameters mentioned above are not included as routine analysis when delivering new batches, the inter-batch variability has not been characterized. When evaluating rheological parameters, inter-batch variability can be due to multiple factors, such as excipient or formulation manufacturing, storage conditions and aging of the formulation.

Daivobet ointment (LeoPharma, Dublin, Ireland) is a combination product of the vitamin D analogue calcipotriol monohydrate and the corticosteroid betamethasone dipropionate. It is indicated for topical treatment of stable plaque *psoriasis vulgaris* in adults. A maximum daily dose of 15 g and maximum body surface area to be treated of 30% should not be exceeded to avoid vitamin D-related adverse events such as hypercalcemia.

The objective of the present work is to assess if the inter-batch variability of the rheological parameters in the reference product Daivobet allows concluding equivalence within a ±10% acceptance range, as defined in the EMA draft guideline on the quality and equivalence of topical products.

## 2. Materials and Methods

### 2.1. Drug Products

Ten batches of Daivobet 50 µg/g + 0.5 mg/g ointment (a two-compound ointment with 50 µg/g calcipotriol and 0.5 mg/g betamethasone dipropionate) were purchased on the Spanish, Portuguese and German markets. The manufacturer of all these batches was Leo Laboratories Ltd. (Dublin, Ireland).

Manufacturing dates ranged from February 2017 to January 2018. Assays were performed between 6 months and 1 year after manufacturing. All samples were preserved at 20–25 °C.

### 2.2. Rheological Analysis

The tests were performed with a controlled stress rheometer Haake^®^ RheoStress^®^1 (Thermo Fisher Scientific, Karlsruhe, Germany) connected to a Haake K10 thermostatic bath, using the RheoWin 4.0 software. After 600 s relaxation time, all measurements (10 batches; 12 replicates per batch) were done at 25 °C using the serrated parallel plates (35 mm diameter, 1 mm gap). All rheological parameters were calculated using the Kaleidagraph 4.03 software (Synergy Software, Reading, PA, USA). The following rheological measurements were performed.

#### 2.2.1. Hysteresis Loops

Hysteresis loops were performed in three stages (rheometer set at controlled shear rate mode):

Upward curve, measuring from 1 s^−1^ and 100 s^−1^ in a stepped ramp (20 steps in logarithmic distribution; 10 s per step).

Continuous rotation at 100 s^−1^ for 60 s (measuring 10 timepoints).

Downward curve, measuring from 100 s^−1^ to 1 s^−1^ in stepped ramp (20 steps in logarithmic distribution; 10 s per step).

The relative thixotropic areas were obtained using Equation (1). The areas enclosed by both the upward (S_A_) and the downward (S_D_) curves were calculated by numerical integration, the difference between them being the thixotropic area (S_T_). The relative thixotropic area (S_R_) [5] is useful in order to compare different samples:

(1) $$S_{R}\left(\%\right)=\frac{S_{A}\;-S_{D}}{S_{A}}\times 100$$

#### 2.2.2. Flow Curves

Stepped flow curves were performed in controlled stress mode in logarithmic distribution with 20 steps and 60 s per step. Stress ranged between 1 Pa (initial σ) and 1300 Pa (final σ). The simplified Carreau model was satisfactorily fitted to viscosity values as a function of shear rate data [6]:

(2) $$\eta =\frac{\eta_{0}}{{\left[1+{\left(\frac{\dot{\gamma }}{{\dot{\gamma }}_{c}}\right)}^{2}\right]}^{S}}$$

where *η_0_* is the zero-shear viscosity, $\dot{\gamma }$_c_ is critical shear rate, and *s* the shear thinning index. Viscosity corresponding to 100 s^−1^ was also calculated from the curve fits obtained.

Yield stress (σ_0_) was estimated on the double log scaled rheograms *η =* *f* *(σ)* from the point where the straight line corresponding to the viscosity plateau intersects the tangent line to the fall in viscosity [7].

#### 2.2.3. Viscoelastic Properties from Oscillatory Tests

The elastic (*G’*) and viscous (*G″*) moduli were obtained from the frequency sweep tests performed between 0.01 and 10 Hz at a stress amplitude of 10 Pa (within the linear viscoelastic region, previously determined from stress sweep tests at 1 Hz).

The dynamic spectra, i.e., the variation of viscoelastic moduli as a function of oscillation frequency *ν* (in Hz) were fitted to power law empirical equations:

(3) $$G^{\prime }=G_{1}^{\prime }\cdot^{\nu^{m^{\prime }}}$$

(4) $$G^{\prime \prime }=G_{1}^{\prime \prime }\cdot^{\nu^{m^{\prime \prime }}}$$

where $G_{1}^{\prime }$ and $G_{1}^{\prime \prime }$ are the calculated moduli at a frequency of 1 Hz and *m’* and *m″* (dimensionless) correspond to the slopes in double logarithmic scales, indicating the frequency-dependence of each modulus [8].

The tangent of the phase shift, or loss tangent, which gives the relation between both dynamic moduli,

(5) $$tan\;\delta =\;\frac{G^{\prime \prime }}{G^{\prime }}$$

was also calculated at 1 Hz.

### 2.3. Spreadability Measurements

Spreadability was calculated by placing 1 g of sample between two glass dishes and progressively adding 50, 100 and 200 g weights on top (10 batches; 12 replicates per batch). Weight was removed after 5 min and the diameter of the squeezed product was measured. The surface area versus weight curve was calculated using Excel 2016 (Microsoft, Redmond, WA, USA).

### 2.4. Parametric Comparison

The physical parameters of the 10 batches (12 replicates each) were compared by three different methods (see below) using Excel 2016 (Microsoft) and assuming homoscedasticity.

#### 2.4.1. Comparison of 1 Batch vs. 1 Batch

The physical parameters of each batch of the reference product were compared with those of the other nine batches (45 possible combinations), assessing whether they would be considered equivalent. Equivalence was considered when the 90% CI of the ratio between batches being compared was within a certain acceptance range. The frequency of considering equivalence was determined at different acceptance limits ranging from ±10% to ±30% (i.e., from 90.00–111.11% to 70.00–142.86%). When at least 80% of comparisons were within the limit evaluated, the parameters were considered equivalent.

#### 2.4.2. Comparison of 5 Batches vs. 5 Batches

The physical parameters of five out of 10 batches were selected as test product and compared with the other five batches as reference product (126 possible combinations). Equivalence was considered when the 90% CI of the ratio between mean of five batches of test and reference products being compared were within a certain acceptance range. The frequency of considering equivalence was calculated as described above.

#### 2.4.3. Comparison of Median of 5 Batches vs. Median of 5 Batches

Again five out of 10 batches were selected as test product and compared with the other five batches as reference product (126 possible combinations). To compare each physical parameter, the mean value of the median batch within each group were considered. The frequency of considering equivalence was calculated as described above.

### 2.5. Non-Parametric Comparison

Non-parametric comparisons were done using bootstrap [9]. Analyses were conducted using experimental data of these physical parameters (described in Section 0 and 0). Ninety percent non-parametric CI were calculated from the probability distribution of the geometric mean ratios from 10,000 bootstraps. For each simulation, a reference and a test batch were randomly selected without replacement. The 90% CI estimation was assessed by either a “1 vs. 1″ comparison (one reference vs. one test batch) or a “5 vs. 5″ comparison (five reference vs. five test batches). All these bootstrap analyses were performed using either 6, 12, or 24 replicates of each parameter, randomly sampled with replacement. Similarity was concluded when the non-parametric 90% CI for the ratio test/reference was within the ±10% acceptance range (90.00–111.11%). R (V. 3.5.1) and R-studio (V.1.1.447) were used for the bootstrap analyses and graphical display, respectively.

## 3. Results and Discussion

The rheological study of the Daivobet formulation demonstrated its pseudoplastic behavior (Supplementary Figure S1). Consequently, various physical properties of 10 batches (12 replicates each) were characterized by calculating relative thixotropic area (*S_R_*), yield stress (*σ_0_*), zero shear viscosity (*η_0_*), viscosity al 100 s^−1^ (*η_100_*), loss tangent (*tan* *δ*), calculated elastic and viscous moduli at 1 Hz ($G_{1}^{\prime }$ and $G_{1}^{\prime \prime }$, respectively), the parameters *m’* and *m″* of the fit, and spreadability (Table 1). Raw data are shown in Table S1.

Yield stress (i.e., the minimum force required by a formulation to flow) and the viscosity of the sample at low shear rates (*η_0_*), relate to the area occupied when applying weight on the formulation. This area is obtained through spreadability measurements, which are simple routine tests for semisolid preparations informing about the spreadability of a formulation when applied on skin, while the other two parameters mentioned correspond to a more rigorous analysis of flow properties. The internal structure of formulations and their viscoelastic properties is given by the determination of storage and loss moduli (*G’* and *G″*) and their dependence with frequency oscillation. In the here tested formulation, elastic behaviour clearly predominated over viscous (*G’* > *G″*) and both moduli varied with frequency, as characterized by their slopes (i.e., *m’* and *m″*) in double logarithmic scale.

For all parameters tested, total variability, measured as coefficient of variation (CV) from all replicates of all batches (*n* = 120), was <15%. The parameters *η_100_, tan δ*, *m’*, *m″* and spreadability showed an inter-batch CV ≤ 5.7%. The contribution to the total variability of each parameter was similar in terms of inter- and intra-batch variability.

**Figure 1:**
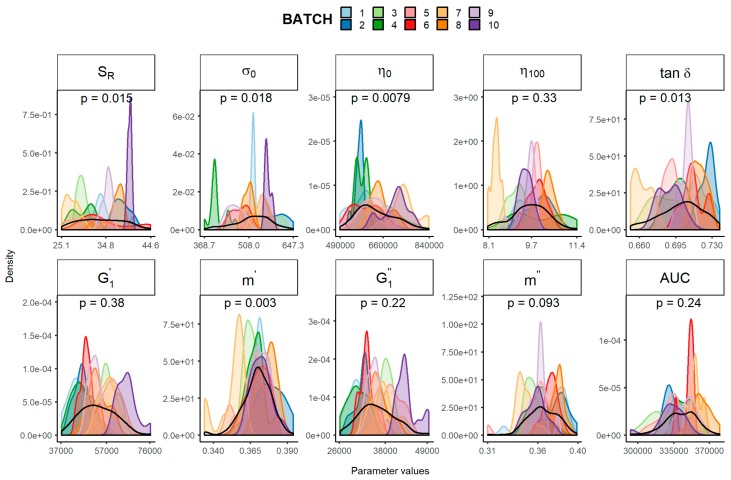
Experimental distribution of each batch (coloured) and overall distribution (black) stratified by the rheological parameters considered. The *p* value of the Shapiro–Wilk test assesses the normality of the overall distribution. *S_R_*, relative thixotropic area; *σ_0_,* yield stress; *η_0_*, zero-shear viscosity; *η_100_*, viscosity at 100 s^−1^; *tan δ*, loss tangent at 1 Hz; $G_{1}^{\prime }$, calculated elastic modulus; $G_{1}^{\prime \prime }$, calculated viscous modulus; *m’* and *m*″ are the parameters obtained when fitting G’ and G’’, respectively, versus frequency; AUC, area under the surface versus weight curve.

To evaluate whether these physical parameters followed a normal distribution, the Shapiro–Wilk test [10,11] was performed. As shown in Figure 1, the experimental distribution of these ten batches were notably different. Based on the *p* value of the Shapiro–Wilk test, neither *S_R_*, *σ_0_*, *η_0_*, *tan* *δ* nor *m’* follow a normal distribution. Those physical parameters not following a normal distribution would not qualify for comparison according to the EMA draft guideline [4].

Next, parametric comparison was conducted using three different calculation methods: comparing one batch vs. one batch, comparing the mean of five batches vs. the mean of five batches and comparing the median batch within five batches vs. the median batch within five batches (Table 2). The values in bold identify the acceptance range that provides the conclusion of equivalence in at least 80% of the comparisons. For those in vitro parameters with low inter-batch variability below 3.7%, (*tan* *δ, m’, m″* and spreadability), the equivalence with a ±10% acceptance range for the 90% CI of the test/reference ratio could be concluded in ≥80% of comparisons, regardless of the comparison method used. For parameters with inter-batch CV around 5.7% (*η_100_*), the number of successful comparisons was 100% if the number of batches compared was 5 either comparing mean values or the median batch.

However, for those parameters with high inter-batch variability (CV ≥ 9.6%; *S_R_,* *σ_0_, η_0_,* $G_{1}^{\prime }$ and $G_{1}^{\prime \prime }$) the equivalence between batches of the same reference product could not be concluded in more than 80% of comparisons, by any of the comparison methods. Thus, according to the EMA draft guideline these batches of same reference formulation would not be considered equivalent in half of the physical parameters evaluated [4], despite the fact that more than the minimum required number of batches (five instead of only three) were tested. Since the equivalence criterion seems to be inappropriate when inter-batch variability is large, the acceptance range could be widened under these circumstances.

When widening the acceptance range to ±15%, a range applied for orally inhaled products [12], equivalence could be concluded in ≥80% of comparisons for almost all parameters (Table 2). The only parameter for which equivalence could not be concluded was *S_R_*, which with 79% successful comparisons was very close of being considered equivalent.

The frequency in which equivalence could be concluded varied between comparison methods. The comparison of the population means (“5 vs. 5 batches″ method) exhibited a higher success percentage than the comparison of individual batches (“1 vs. 1 batch″ method) or than the comparison of the batches with the median values within five batches (“median batch vs. median batch″) (Table 2). With a 15% acceptance range, for instance, equivalence for $G_{1}^{\prime }$, the parameter with the highest inter-batch variability, could be concluded in 42%, 86% and 62% of cases with the “1 vs. 1″, “5 vs. 5″ and the “median batch vs. median batch″ method, respectively.

Selection of the most representative batch for comparison, as done when comparing the median batch within five batches vs. the median batch within five batches, is required for in vivo studies [13], as evaluation of more than one batch is not feasible. According to the data here presented, this approach would not be appropriate for in vitro equivalence evaluations, while the comparison of means, as proposed by the EMA [4], is the preferable method.

Using conventional sample size calculation, a sample size of 56 per group would provide 80% power to conclude equivalence within an acceptance range of ±10%, assuming a difference between test and reference of 5% and an inter-individual CV of 12%. However, as the data here presented show, an even larger sample size (five batches per group and 12 replicates per batch, i.e., 60 samples per group) concluded equivalence in less than 80% (76%) of the comparisons between groups of 5 batches (Table 2) for a rheological parameter (*η_0_*) with a total CV of 11.8% (and an inter-batch CV of 9.6%) (Table 1) and a theoretical difference between batches of zero (batches of the same reference product). Therefore, a sample size calculation taking into account inter-batch variability would be necessary to ensure that the experiments have the desired power. In the absence of proper sample size calculations, pharmaceutical companies might be tempted to conduct pilot experiments to select those batches that behave similarly, before performing a formal comparison for regulatory submission, which could be considered data manipulation.

Since the parametric comparison relies on normality assumption of the evaluated parameters, a requirement not met by most of the rheological parameters here evaluated, a bootstrap analysis was performed. Bootstrap methodology is currently used in several disciplines including drug research, where it has improved the comparison of dissolution formulations under high variability conditions [14], as well as the evaluation of population pharmacokinetic/pharmacodynamic models [15,16,17]. Based on the percentile distribution of the geometric means, the bootstrap methodology randomly samples batches (without replacement) and replicates to construct a non-parametric CI. Thus, this methodology does not require assumption of normal distribution.

**Figure 2:**
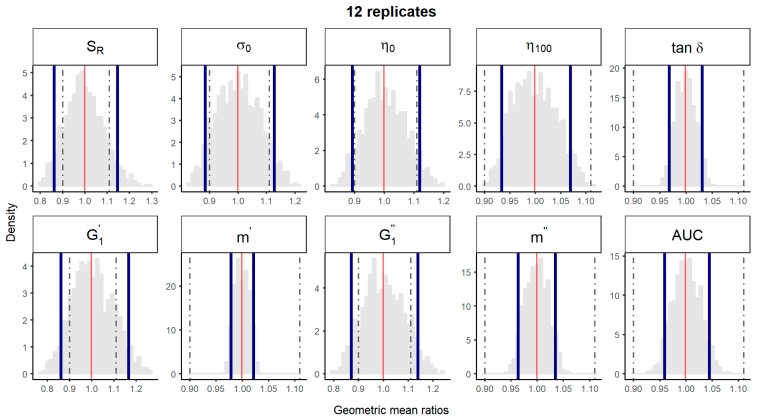
Bootstrap analysis of rheological parameters - 1 reference batch versus 1 reference batch. 10,000 geometric mean ratios (light grey area) resulting from the bootstrap analysis of “1 reference batch *versus* 1 test batch″ for each rheological parameter. Data of 10 batches and 12 replicate each were used. Median (red line) and non-parametric 90% CI (blue lines) of the probability distribution. Dashed lines represent the acceptance limits for equivalence (90–111.11%) stated in the EMA guideline [4]. *S_R_*, relative thixotropic area; *σ_0_,* yield stress; *η_0_*, zero-shear viscosity; *η_100_*, viscosity at 100 s^−1^; *tan δ*, loss tangent;$\;G_{1}^{\prime }$, calculated elastic modulus; $G_{1}^{\prime \prime }$, calculated viscous modulus; *m’* and *m*″ are the parameters obtained when fitting G’ and G’’, respectively, versus frequency; AUC, area under the surface versus weight curve (spreadability).

**Figure 3:**
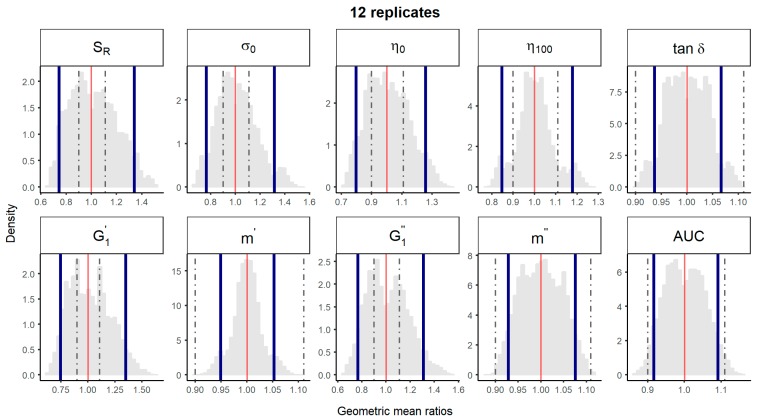
Bootstrap analysis of rheological parameters – five reference batches versus five reference batches. 10,000 geometric mean ratios (light grey area) resulting from the bootstrap analysis of “5 reference batches *versus* five test batches″ for each rheological parameter. Data of 10 batches and 12 replicate each were used. Median (solid red line) and non-parametric 90% CI (solid blue lines) of the probability distribution. Dashed lines represent the acceptance limits for equivalence (90–111.11%) stated in the EMA guideline [4]. *S_R_*, relative thixotropic area; *σ_0_,* yield stress; *η_0_*, zero-shear viscosity; *η_100_*, viscosity at 100 s^−1^; *tan δ*, loss tangent at 1 Hz;$\;G_{1}^{\prime }$, calculated elastic modulus; $G_{1}^{\prime \prime }$, calculated viscous modulus; *m’* and *m*″ are the parameters obtained when fitting G’ and G’’, respectively, versus frequency; AUC, area under the surface versus weight curve (spreadability).

A bootstrap analysis of the physical parameters was performed using the experimental data described above. The 10,000 geometric mean ratios with 12 replicates and non-parametric 90% CI were represented, using either the “1 batch vs. 1 batch″ (Figure 2) or the “5 batches vs. 5 batches″ (Figure 3) comparison method. Non-parametric 90% CI of physical parameters *tan* *δ, m’, m″* and spreadability laid within the ±10% limits irrespective of the method of comparison. Thus, equivalence among batches could be concluded for parameters with total variability <5%. However, for parameters with total CV between 5 and 10% (i.e., *η_100_*), equivalence could only be concluded with the “5 vs. 5″ comparison method, the probability distributions of the geometric means of the “1 vs. 1″ comparison method being notably wider (Figure 2 and Figure 3). For rheological parameters with total CV > 10% *(S_R_,* *σ_0_, η_0_,* $G_{1}^{\prime }$ and $G_{1}^{\prime \prime }$), equivalence could not be concluded by neither the “1 vs. 1″, nor the “5 vs. 5″ method.

Next, the impact of number of replicates on the bootstrap analysis was evaluated. The conclusion regarding confirmation of equivalence was independent on whether 6, 12 or 24 replicates were used (Supplementary Figures S2, S3, S4 and S5). The distribution values were quite similar and had no impact on the 90% CI estimation. Altogether, these data indicate that 6 replicates are enough to conclude equivalence by bootstrap methodology. For physical parameters with high inter-batch variability, the acceptance limit of ±10% was however too strict to permit conclusion of equivalence between batches of the same reference product.

To sum up, the same conclusions on equivalence could be drawn with either parametric and non-parametric analysis, using 12 or six replicates, respectively. Equivalence in microstructure between batches of the same reference drug for topical use could not be demonstrated for most of the parameters tested, when following the EMA draft guideline for comparison of test and reference drugs for topical use [4]. According to this guideline, the 90% CI for the difference of means of the test and comparator products should be contained within the acceptance range of ±10% of the comparator product mean, assuming normal distribution of data, with at least three batches of test product and three batches of reference product and at least 12 replicates per batch [4]. When comparing individual batches, equivalence could only be concluded for those parameters with a total CV < 5%. When comparing the median values of five batches, equivalence could only be concluded for parameters with a total CV < 10%. If the comparisons of the means had been done with only three batches, strictly following the minimum requirement of the EMA guideline, equivalence would only be concluded for parameters with less than 5.7% inter-batch variability and, consequently, fewer microstructure parameters would have been considered equivalent.

Altogether, the data here presented suggest that an acceptance range of ±10% to conclude equivalence of microstructure parameters of semisolid dosage forms [4] is too strict, given the high inter-batch difference observed between batches of the same reference product. Following approaches could overcome this handicap: (1) a sample size calculation taking into account the inter-batch variability, to ensure that the number of batches is appropriate to obtain the desired power, or (2) widening the limits to ±15%, since this acceptance range seems to be suitable when the products are supposed to be the same (Table 2 and Figure 3), or to ±20%, which is the conventional acceptance range for AUC and C_max_ in pharmacokinetic bioequivalence studies [18] and it is also proposed by other authors for the comparison of some in vitro parameters [19].

## 4. Conclusions

Widening of the ±10% acceptance range based on the inter-batch variability of the reference product seems to be justified when following the methodology and sample size proposed in the EMA draft guideline. Otherwise, the sample size calculation should consider the inter-batch variability, which could render an excessive number of batches to be tested. In our opinion, in vitro testing of more than 5 batches each of test and reference product might be excessive for the development of an abbreviated topical product with a qualitative and quantitative composition equivalent to the reference product.

## Figures and Tables

**Table 1:** Physical parameters (rheological properties and spreadability) of 10 batches (12 replicates each) of reference formulation.

Parameter	Mean	SD	Minimum	Maximum	Total CV (%)	Inter-Batch CV (%)	Intra-Batch CV	
							Minimum	Maximum
***S_R_* (%)**	33.80	4.82	25.06	44.56	14.3	12.2	1.0	16.4
***σ_0_* (Pa)**	519	57	369	647	11.0	10.6	1.6	6.8
***η_0_* (Pa·s)**	630,067	74,229	488,890	839,980	11.8	9.6	3.6	10.1
***η_100_* (Pa·s)**	9.63	0.67	8.05	11.40	7.0	5.7	2.0	9.3
***tan δ***	0.700	0.020	0.651	0.737	2.9	2.7	0.8	2.0
$G_{1}^{\prime }$ **(Pa)**	53,255	7741	37,195	76,270	14.6	13.1	5.9	11.5
***m′***	0.369	0.010	0.334	0.394	2.6	1.9	1.3	2.7
$G_{1}^{\prime \prime }$ **(Pa)**	35,829	4723	26,149	49,457	13.2	11.7	5.2	10.6
***m″***	0.365	0.015	0.310	0.399	4.1	3.1	1.8	4.6
**Spreadability (mm^2^)**	342,224	15,438	292,922	379,991	4.5	3.7	1.7	4.9

CV, coefficient of variation; SD, standard deviation. *S_R_*, relative thixotropic area; *σ_0_*, yield stress; *η_0_,* zero-shear viscosity; *η_100_,* viscosity at 100 s^−1^; *tan δ*, loss tangent measured at 1 Hz; $G_{1}^{\prime }$, calculated elastic modulus; $G_{1}^{\prime \prime }$, calculated viscous modulus; *m′* and *m″* are the parameters obtained when fitting *G′* and *G″*, respectively, *versus* frequency.

**Table 2:** Comparison of rheological parameters and spreadability. Number of ratios within the limits of equivalence (*n*) divided by total number of comparisons made (*N*).

Comparison Method	1 Batch vs. 1 Batch					5 Batches vs. 5 Batches				Median Batch within 5 Batches vs. Median Batch within 5 Batches			
Acceptance Range	10%	15%	20%	25%	30%	10%	15%	20%	25%	10%	15%	20%	25%
Parameter													
***S_R_* (%)**	7/45 (16%)	17/45 (38%)	27/45 (60%)	**36/45 (80%)**	43/45 (96%)	66/126 (52%)	100/126 (79%)	**124/126 (98%)**	126/126 (100%)	54/126 (43%)	60/126 (48%)	**105/126 (83%)**	126/126 (100%)
***σ_0_* (Pa)**	17/45 (38%)	26/45 (58%)	34/45 (76%)	**39/45 (87%)**	42/45 (93%)	83/126 (66%)	**120/126 (95%)**	126/126 (100%)	126/126 (100%)	90/126 (71%)	**111/126 (88%)**	126/126 (100%)	126/126 (100%)
***η _0_* (Pa·s)**	10/45 (22%)	25/45 (56%)	**36/45 (80%)**	41/45 (91%)	45/45 (100%)	96/126 (76%)	**124/126 (98%)**	126/126 (100%)	126/126 (100%)	**0/126 (0%)**	**102/126 (81%)**	117/126 (93%)	126/126 (100%)
***η _100_* (Pa·s)**	33/45 (73%)	**39/45 (87%)**	44/45 (98%)	45/45 (100%)	45/45 (100%)	**126/126 (100%)**	126/126 (100%)	126/126 (100%)	126/126 (100%)	**126/126 (100%)**	126/126 (100%)	126/126 (100%)	126/126 (100%)
***tan δ ***	**45/45 (100%)**	45/45 (100%)	45/45 (100%)	45/45 (100%)	45/45 (100%)	**126/126 (100%)**	126/126 (100%)	126/126 (100%)	126/126 (100%)	126/126 (100%)	126/126 (100%)	126/126 (100%)	126/126 (100%)
$G_{1}^{\prime }$ **(Pa)**	7/45 (16%)	19/45 (42%))	25/45 (56%)	34/45 (76%)	**42/43 (93%)**	65/126 (52%)	**108/126 (86%)**	124/126 (98%)	126/126 (100%)	36/126 (29%)	78/126 (62%)	84/126 (67%)	**126/126 (100%)**
***m’***	**45/45 (100%)**	45/45 (100%)	45/45 (100%)	45/45 (100%)	45/45 (100%)	**126/126 (100%)**	126/126 (100%)	126/126 (100%)	126/126 (100%)	**126/126 (100%)**	126/126 (100%)	126/126 (100%)	126/126 (100%)
$G_{1}^{\prime \prime }\;$ **(Pa)**	7/45 (16%)	20/45 (44%)	31/45 (69%)	**39/45 (87%)**	43/45 (96%)	76/126 (60%)	**117/126 (93%)**	126/126 (100%)	126/126 (100%)	36/126 (29%)	84/126 (67%)	**126/126 (100%)**	126/126 (100%)
***m″***	**45/45 (100%)**	45/45 (100%)	45/45 (100%)	45/45 (100%)	45/45 (100%)	**126/126 (100%)**	126/126 (100%)	126/126 (100%)	126/126 (100%)	**126/126 (100%)**	126/126 (100%)	126/126 (100%)	126/126 (100%)
**Spreadability (mm^2^)**	**41/45 (91%)**	45/45 (100%)	45/45 (100%)	45/45 (100%)	45/45 (100%)	**126/126 (100%)**	126/126 (100%)	126/126 (100%)	126/126 (100%)	**126/126 (100%)**	126/126 (100%)	126/126 (100%)	126/126 (100%)

Data are presented as *n*/*N* (%), n being the number of ratios within the respective limit of equivalence and *N* being the total number of comparisons made. Numbers in **bold** identify the lowest acceptance range that concludes equivalence ≥ 80% of comparisons. *S_R_*, relative thixotropic area; *σ_0_,* yield stress; *η_0_*, zero-shear viscosity; *η_100_*, viscosity at 100 s^−1^; *tan δ*, loss tangent at 1 Hz; $G_{1}^{\prime }$, calculated elastic modulus; $G_{1}^{\prime \prime }$, calculated viscous modulus; *m′* and *m*″ are the parameters obtained when fitting G′ and G″, respectively, *versus* frequency.

## Supplementary Materials

The following are available online at https://www.mdpi.com/1999-4923/11/10/503/s1: Figure S1: Representative rheograms of evaluated batches. Figure S2: Bootstrap analysis of rheological parameters using 6 replicates - 1 reference batch versus 1 reference batch. Figure S3: Bootstrap analysis of rheological parameters using 24 replicates - 1 reference batch versus 1 reference batch. Figure S4: Bootstrap analysis of rheological parameters using 6 replicates – 5 reference batches versus 5 references batches. Figure S5: Bootstrap analysis of rheological parameters using 24 replicates – 5 reference batches versus 5 references batches. Table S1: Raw data.
