# Erratum: Mangas-Sanjuán, V.; et al. Assessment of the Inter-Batch Variability of Microstructure Parameters in Topical Semisolids and Impact on the Demonstration of Equivalence. *Pharmaceutics* 2019, *11*, 503

**DOI:** 10.3390/pharmaceutics12050436

**Published:** 2020-05-09

**Authors:** Víctor Mangas-Sanjuán, María Pleguezuelos-Villa, Matilde Merino-Sanjuán, Mᵃ Jesús Hernández, Amparo Nácher, Alfredo García-Arieta, Daniel Peris, Irene Hidalgo, Lluís Soler, Marta Sallan, Virginia Merino

**Affiliations:** 1 Departamento de Farmacia y Tecnología Farmacéutica y Parasitología, Facultad de Farmacia, Universitat de València, Av. Vicente Andrés Estellés s/n, Burjassot, 46100 Valencia, Spain; victor.mangas@uv.es (V.M.-S.); maplevi@alumni.uv.es (M.P.-V.); matilde.merino@uv.es (M.M.-S.); amparo.nacher@uv.es (A.N.); 2 Instituto Interuniversitario de Investigación de Reconocimiento Molecular y Desarrollo Tecnológico (IDM), Universitat Politècnica de València, Universitat de València, 46100 Valencia, Spain; 3 Departament de Fisica de la Terra i Termodinàmica, Universitat de València, Vicente Andrés Estelles s/n. Burjassot, 46100 Valencia, Spain; M.Jesus.Hernandez@uv.es; 4 División de Farmacología y Evaluación Clínica, Departamento de Medicamentos de Uso Humano, Agencia Española de Medicamentos y Productos Sanitarios, Calle Campezo 1, Ed 8, 28022 Madrid, Spain; agarciaarieta@gmail.com; 5 Pharmacokinetics and Clinical Affairs Department, Strategy and Development Area, Kern Pharma S.L., Calle Venus 72, Terrassa, 08228 Barcelona, Spain; danielperis@outlook.es (D.P.); ihidalgom@kernpharma.com (I.H.); 6 Formulation and Late Scale Development Department, Strategy and Development Area, Kern Pharma S.L., Calle Venus 72, Terrassa, 08228 Barcelona, Spain; lsoler@kernpharma.com (L.S.); msallan@kernpharma.com (M.S.)

The authors wish to make the following corrections to this paper [1]: Figures 2 and 3 captions have been inadvertently interchanged. Figures S2 and S4 captions have been inadvertently interchanged. Figures S3 and S5 captions have been inadvertently interchanged. Figures 2, 3 and S2–S5 have now been corrected in this erratum.

The authors would like to apologize for any inconvenience caused to the readers by these changes.

**Figure 2:**
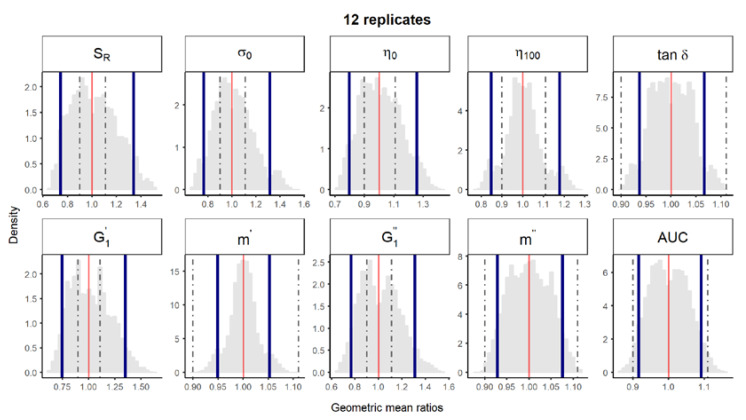
Bootstrap analysis of rheological parameters—1 reference batch versus 1 reference batch. A total of 10,000 geometric mean ratios (light grey area) resulting from the bootstrap analysis of “1 reference batch versus 1 test batch” for each rheological parameter. Data of 10 batches and 12 replicates each were used. Median (red line) and non-parametric 90% CI (blue lines) of the probability distribution. Dashed lines represent the acceptance limits for equivalence (90–111.11%) stated in the EMA guideline [4]. *S_R_*, relative thixotropic area; *σ_0_,* yield stress; *η_0_*, zero-shear viscosity; *η_100_*, viscosity at 100 s^−1^; *tan δ*, loss tangent;$\;G_{1}^{\prime }$, calculated elastic modulus; $G_{1}^{\prime \prime }$, calculated viscous modulus; *m’* and *m*” are the parameters obtained when fitting G’ and G”, respectively, versus frequency; AUC, area under the surface versus weight curve (spreadability).

**Figure 3:**
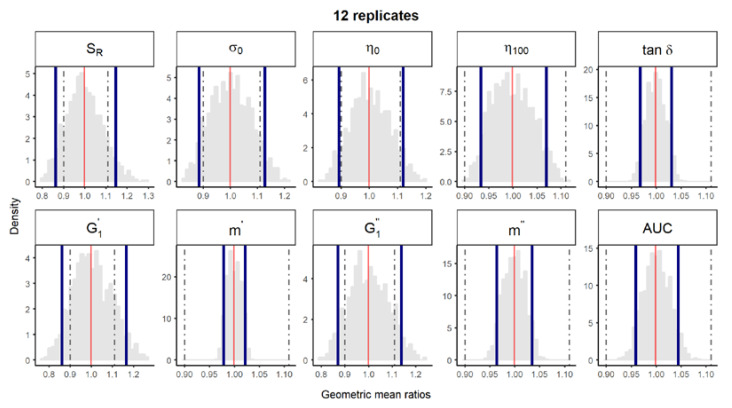
Bootstrap analysis of rheological parameters—five reference batches versus five reference batches. A total of 10,000 geometric mean ratios (light grey area) resulting from the bootstrap analysis of “5 reference batches versus five test batches” for each rheological parameter. Data of 10 batches and 12 replicate each were used. Median (solid red line) and non-parametric 90% CI (solid blue lines) of the probability distribution. Dashed lines represent the acceptance limits for equivalence (90–111.11%) stated in the EMA guideline [4]. *S_R_*, relative thixotropic area; *σ_0_,* yield stress; *η_0_*, zero-shear viscosity; *η_100_*, viscosity at 100 s^−1^; *tan δ*, loss tangent at 1 Hz;$\;G_{1}^{\prime }$, calculated elastic modulus;$\;G_{1}^{\prime \prime }$, calculated viscous modulus; *m’* and *m*” are the parameters obtained when fitting G’ and G”, respectively, versus frequency; AUC, area under the surface versus weight curve (spreadability).

**Figure S2:**
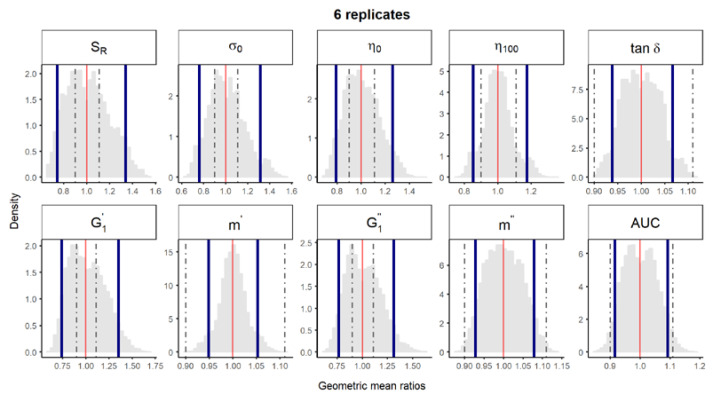
Bootstrap analysis of rheological parameters using 6 replicates—1 reference batch versus 1 reference batch. A total of 10,000 geometric mean ratios (light grey area) resulting from the bootstrap analysis of “1 reference batch versus 1 test batch” for each rheological parameter. Data of 10 batches and 6 replicate each were used. Median (red line) and non-parametric 90% CI (blue lines) of the probability distribution. Dashed lines represent the acceptance limits for equivalence (90–111.11%) stated in the EMA guideline [4]. *S_R_*, relative thixotropic area; *σ_0_*, yield stress; *η_0_*, zero-shear viscosity; *η_100_*, viscosity at 100 s^−1^; *tan δ*, loss tangent at 1 Hz; $\;G_{1}^{\prime }$, calculated elastic modulus; $G_{1}^{\prime \prime }$, calculated viscous modulus; *m’* and *m*” are the parameters obtained when fitting *G’* and *G”*, respectively, versus frequency; *AUC*, area under the weight versus surface curve (spreadability).

**Figure S3:**
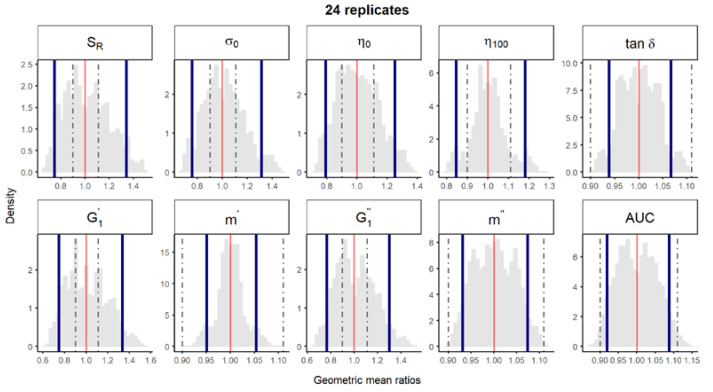
Bootstrap analysis of rheological parameters using 24 replicates—1 reference batch versus 1 reference batch. A total of 10,000 geometric mean ratios (light grey area) resulting from the bootstrap analysis of “1 reference batch versus 1 test batch” for each rheological parameter. Data of 10 batches and 24 replicate each were used. Median (red line) and non-parametric 90% CI (blue lines) of the probability distribution. Dashed lines represent the acceptance limits for equivalence (90–111.11%) stated in the EMA guideline [4]. *S_R_*, relative thixotropic area; *σ_0_*, yield stress; *η_0_*, zero-shear viscosity; *η_100_*, viscosity at 100 s^−1^; *tan δ*, loss tangent at 1 Hz; $\;G_{1}^{\prime }$, calculated elastic modulus; $G_{1}^{\prime \prime }$, calculated viscous modulus; *m’* and *m*” are the parameters obtained when fitting *G’* and *G”*, respectively, versus frequency; *AUC*, area under the weight versus surface curve (spreadability).

**Figure S4:**
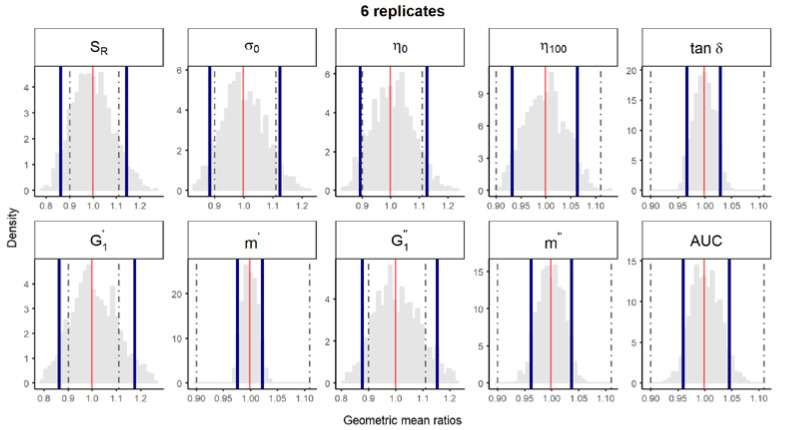
Bootstrap analysis of rheological parameters using 6 replicates—5 reference batches versus 5 references batches. A total of 10,000 geometric mean ratios (light grey area) resulting from the bootstrap analysis of “5 reference batches versus 5 test batches” for each rheological parameter. Data of 10 batches and 6 replicate each were used. Median (solid red line) and non-parametric 90% CI (solid blue lines) of the probability distribution. Dashed lines represent the acceptance limits for equivalence (90–111.11%) stated in the EMA guideline [4]. *S_R_*, relative thixotropic area; *σ_0_*, yield stress; *η_0_*, zero-shear viscosity; *η_100_*, viscosity at 100 s^−1^; *tan δ*, loss tangent at 1 Hz; $\;G_{1}^{\prime }$, calculated elastic modulus; $G_{1}^{\prime \prime }$, calculated viscous modulus; *m’* and *m*” are the parameters obtained when fitting *G’* and *G”*, respectively, versus frequency; *AUC*, area under the weight versus surface curve (spreadability).

**Figure S5:**
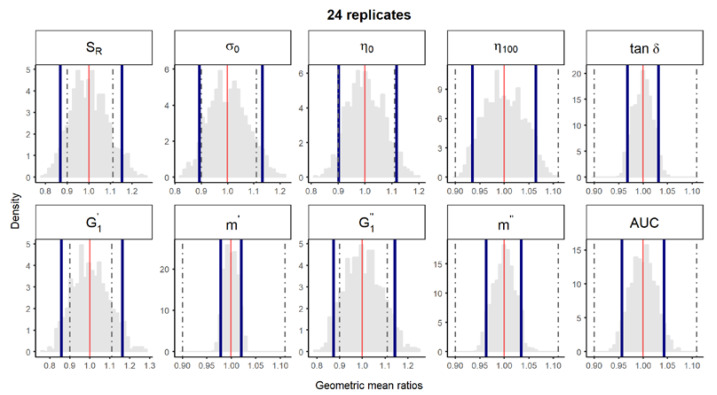
Bootstrap analysis of rheological parameters using 24 replicates—5 reference batches versus 5 references batches. A total of 10,000 geometric mean ratios (light grey area) resulting from the bootstrap analysis of “5 reference batches versus 5 test batches” for each rheological parameter. Data of 10 batches and 24 replicate each were used. Median (solid red line) and non-parametric 90% CI (solid blue lines) of the probability distribution. Dashed lines represent the acceptance limits for equivalence (90–111.11%) stated in the EMA guideline [11]. *S_R_*, relative thixotropic area; *σ_0_*, yield stress; *η_0_*, zero-shear viscosity; *η_100_*, viscosity at 100 s^−1^; *tan δ*, loss tangent at 1 Hz; $\;G_{1}^{\prime }$, calculated elastic modulus; $G_{1}^{\prime \prime }$, calculated viscous modulus; *m’* and *m*” are the parameters obtained when fitting *G’* and *G”*, respectively, versus frequency; *AUC*, area under the weight versus surface curve (spreadability).
