# Supplementary material for: Assessment of the Inter-Batch Variability of Microstructure Parameters in Topical Semisolids and Impact on the Demonstration of Equivalence
Source: Pharmaceutics. 2019 Oct 1;11(10):503. doi: 10.3390/pharmaceutics11100503 (PMC6835722; doi:10.3390/pharmaceutics11100503)
Supplement: Supplementary file 1 [file pharmaceutics-11-00503-s001.pdf]

pharmaceutics

MDPI

Article

Supplementary Materials: Assessment of the Inter-Batch Variability of Microstructure Parameters in Topical Semisolids and Impact on the Demonstration of Equivalence

Víctor Mangas-Sanjuán, María Pleguezuelos-Villa, Matilde Merino-Sanjuán, Mª Jesús Hernández, Amparo Nácher, Alfredo García-Arieta, Daniel Peris, Irene Hidalgo, Lluís Soler, Marta Sallan and Virginia Merino

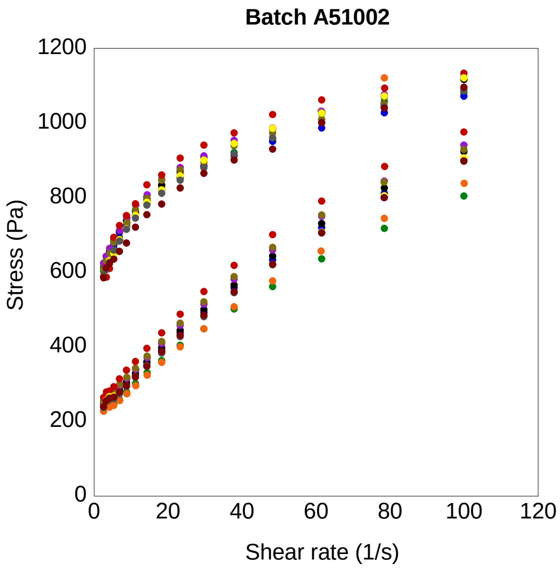

(**A**)

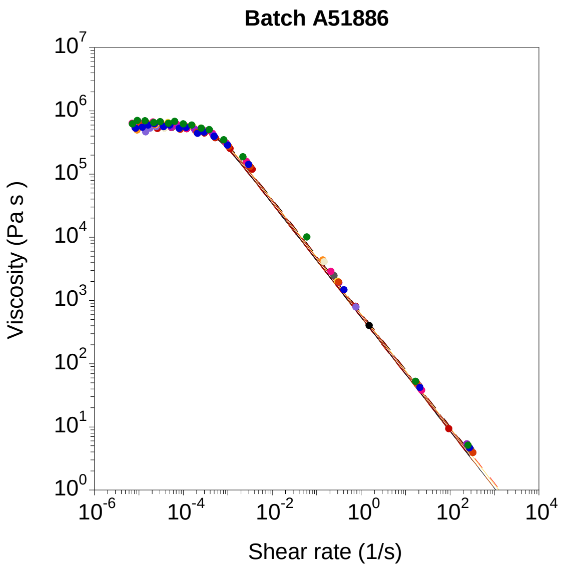

(**B**)

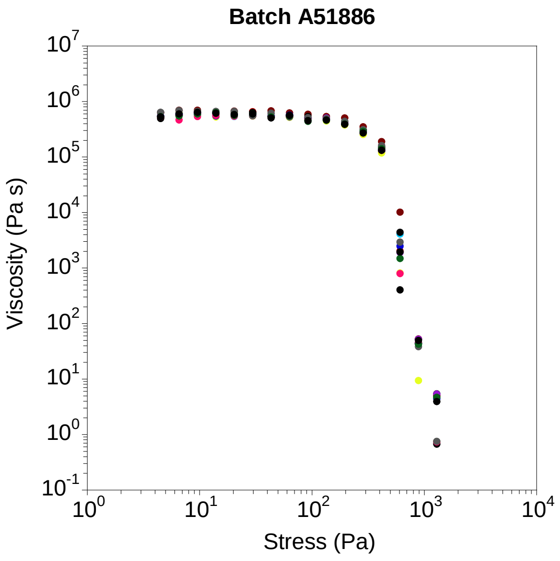

(**C**)

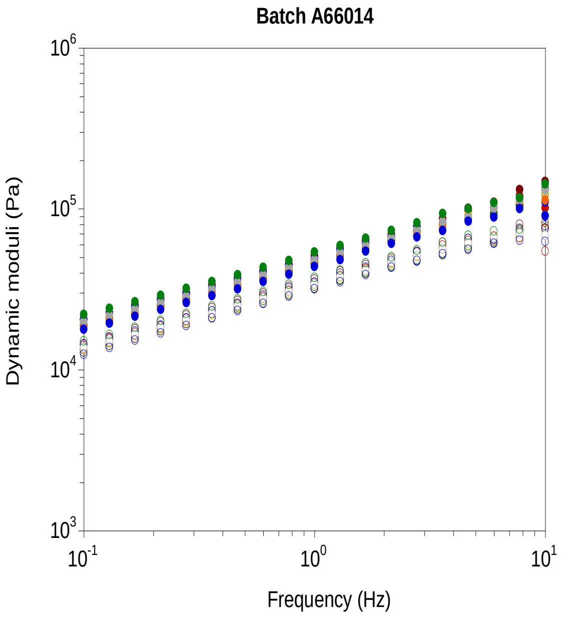

(**D**)

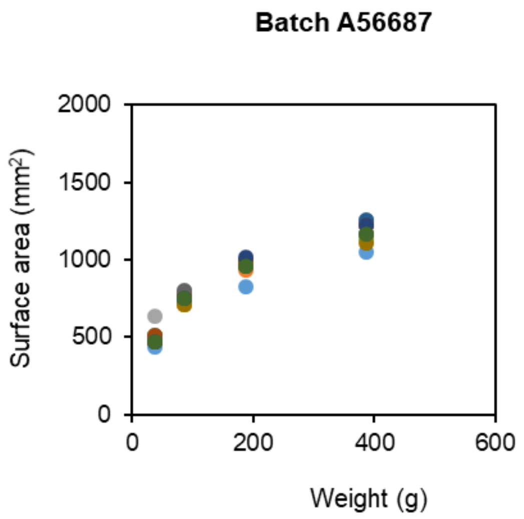

(**E**)

Figure S1. Representative rheograms of evaluated batches. (**A**) Hysteresis cycle. (**B**) Viscosity *versus* shear rate fitted to Carreau model. (**C**) Viscosity versus shear stress. (**D**) Elastic modulus G′ (closed symbols) and viscous modulus G′′ (open symbols) v*ersus* frequency. (**E**) Surface area *versus* weight (spreadability assay).

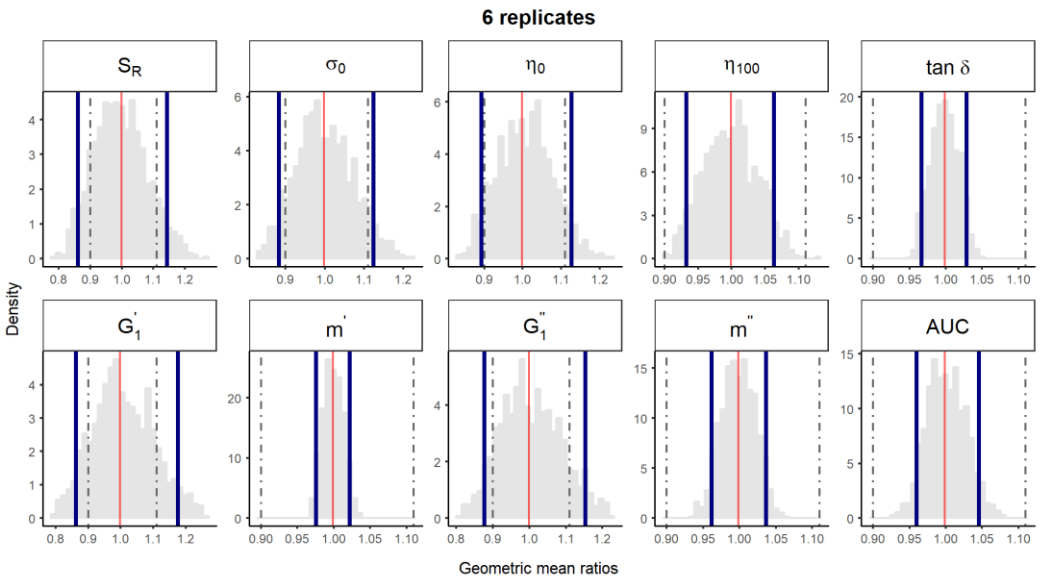

Figure S2. Bootstrap analysis of rheological parameters using 6 replicates - 1 reference batch *versus* 1 reference batch. 10,000 geometric mean ratios (light grey area) resulting from the bootstrap analysis of "1 reference batch *versus* 1 test batch" for each rheological parameter. Data of 10 batches and 6 replicate each were used. Median (red line) and non-parametric 90% CI (blue lines) of the probability distribution. Dashed lines represent the acceptance limits for equivalence (90–111.11%) stated in the EMA guideline [4]. S_R, relative thixotropic area; σ_0, yield stress; η_0, zero-shear viscosity; η_{100}, viscosity at 100 s^{-1}; *tan δ*, loss tangent at 1 Hz; G_1', calculated elastic modulus; G_1'', calculated viscous modulus; m' and m" are the parameters obtained when fitting G' and G'', respectively, *versus* frequency; *AUC*, area under the weight *versus* surface curve (spreadability).

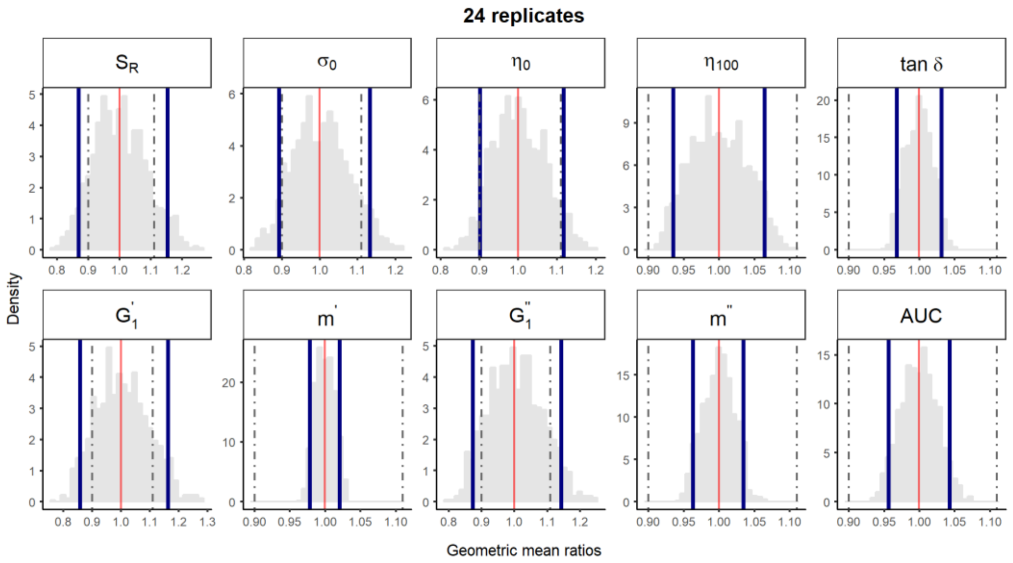

Figure S3. Bootstrap analysis of rheological parameters using 24 replicates - 1 reference batch *versus* 1 reference batch. 10,000 geometric mean ratios (light grey area) resulting from the bootstrap analysis of "1 reference batch *versus* 1 test batch" for each rheological parameter. Data of 10 batches and 24 replicate each were used. Median (red line) and non-parametric 90% CI (blue lines) of the probability distribution. Dashed lines represent the acceptance limits for equivalence (90–111.11%) stated in the EMA guideline [4]. .S_R, relative thixotropic area; σ_0, yield stress; η_0, zero-shear viscosity; η_{100}, viscosity at 100 s^{-1}; *tan δ*, loss tangent at 1 Hz; G_1', calculated elastic modulus; G_1'', calculated viscous modulus; *m′* and m'' are the parameters obtained when fitting *G′* and *G′′*, respectively, *versus* frequency; *AUC*, area under the weight *versus* surface curve (spreadability).

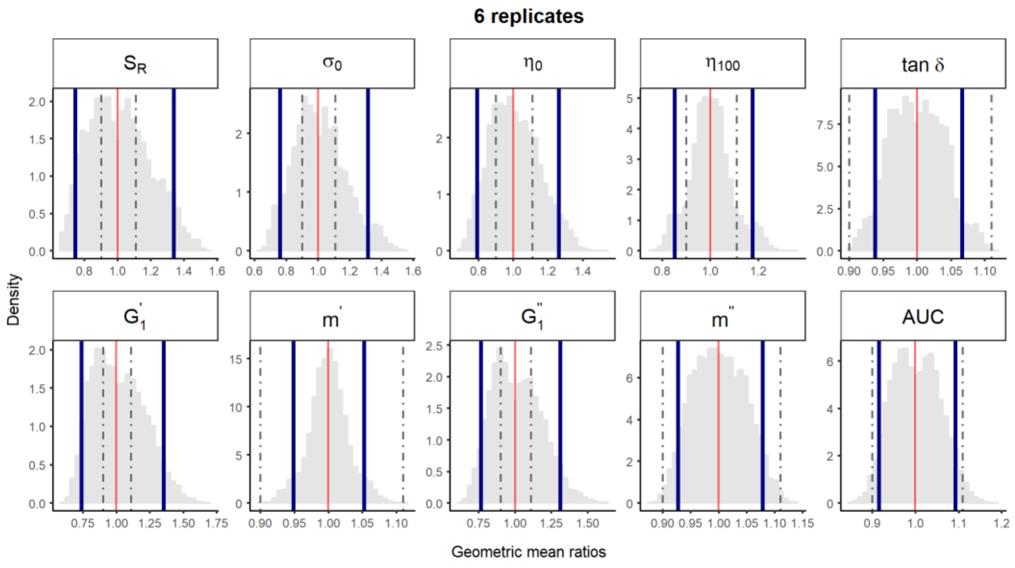

Figure S4. Bootstrap analysis of rheological parameters using 6 replicates – 5 reference batches *versus* 5 references batches.10,000 geometric mean ratios (light grey area) resulting from the bootstrap analysis of "5 reference batches *versus* 5 test batches" for each rheological parameter. Data of 10 batches and 6 replicate each were used. Median (solid red line) and non-parametric 90% CI (solid blue lines) of the probability distribution. Dashed lines represent the acceptance limits for equivalence (90–111.11%) stated in the EMA guideline [4]. .S_R, relative thixotropic area; σ_0, yield stress; η_0, zero-shear viscosity; η_{100}, viscosity at 100 s^{-1}; *tan δ*, loss tangent at 1 Hz; G_1', calculated elastic modulus; G_1'', calculated viscous modulus; *m′* and m'' are the parameters obtained when fitting *G′* and *G′′*, respectively, *versus* frequency; *AUC*, area under the weight *versus* surface curve (spreadability).

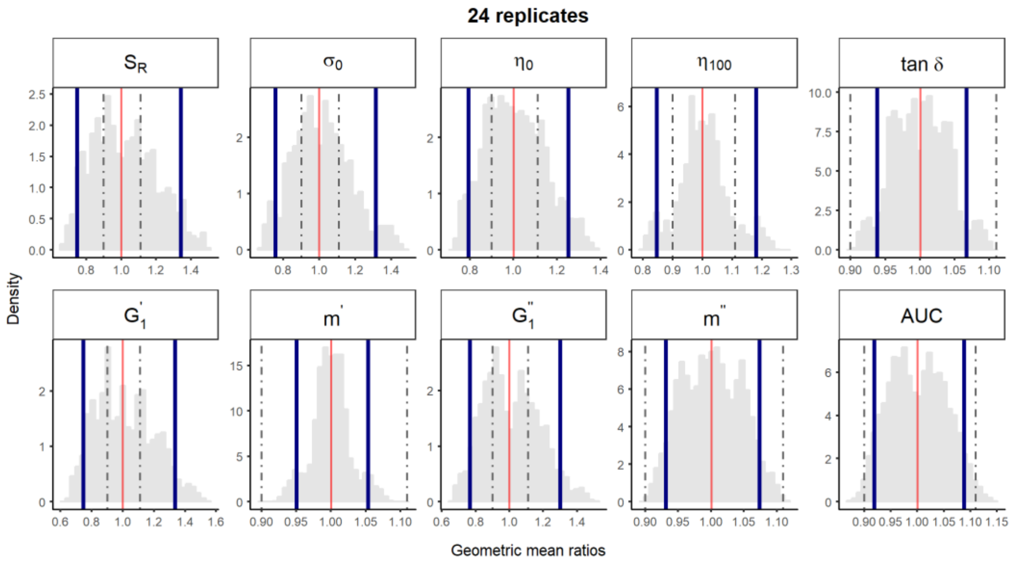

Figure S5. Bootstrap analysis of rheological parameters using 24 replicates – 5 reference batches *versus* 5 references batches. 10,000 geometric mean ratios (light grey area) resulting from the bootstrap analysis of "5 reference batches *versus* 5 test batches" for each rheological parameter. Data of 10 batches and 24 replicate each were used. Median (solid red line) and non-parametric 90% CI (solid blue lines) of the probability distribution. Dashed lines represent the acceptance limits for equivalence (90–111.11%) stated in the EMA guideline [11]. .S_R, relative thixotropic area; σ_0, yield stress; η_0, zero-shear viscosity; η_{100}, viscosity at 100 s^{-1}; *tan δ*, loss tangent at 1 Hz; G_1', calculated elastic modulus; G_1'', calculated viscous modulus; *m′* and m" are the parameters obtained when fitting *G′* and *G′′*, respectively, *versus* frequency; *AUC*, area under the weight *versus* surface curve (spreadability).

Table S1. Raw Data.

Id Batch	Id Replicate	S_R	σ_0	η_0	η_{100}	*tan δ*	G_1'	m'	G_1''	m"	AUC
**1**	1	34.16	489	518980	9.15	0.731	39190	0.372	27080	0.377	351207
**1**	2	33.22	520	542830	9.12	0.711	56684	0.372	38284	0.371	350348
**1**	3	33.52	514	585390	10.09	0.718	47526	0.377	31626	0.326	344349
**1**	4	34.51	527	489920	10.28	0.718	37195	0.366	26149	0.377	338155
**1**	5	35.72	520	572010	9.37	0.713	44185	0.37	30857	0.386	339765
**1**	6	30.76	526	589330	10.19	0.728	47647	0.37	32830	0.378	331616
**1**	7	31.41	522	536160	10.12	0.722	47984	0.376	33804	0.392	334989
**1**	8	32.87	521	603160	9.38	0.713	43664	0.379	30544	0.386	324047
**1**	9	34.07	503	498130	9.12	0.700	42414	0.371	29128	0.382	327655
**1**	10	32.99	521	589440	10.16	0.712	43711	0.371	30485	0.385	337423
**1**	11	40.76	513	614890	9.32	0.734	43244	0.378	29769	0.387	349499
**1**	12	41.01	523	602020	10.25	0.699	40195	0.354	28254	0.373	336609
**2**	1	36.62	647	651010	10.7	0.717	50107	0.37	34997	0.372	349243
**2**	2	36.33	580	557290	10.09	0.727	46522	0.375	32596	0.379	343108
**2**	3	36.92	570	568770	10.08	0.727	47926	0.386	33313	0.382	346195
**2**	4	38.01	603	572200	10.08	0.730	46260	0.358	32850	0.36	337585
**2**	5	36.34	623	621070	10.07	0.721	51402	0.382	36089	0.382	330959
**2**	6	40.56	527	570330	10.49	0.729	55454	0.367	38519	0.364	326359
**2**	7	38.21	535	575350	9.09	0.727	45241	0.394	32022	0.393	326831
**2**	8	39.08	602	573150	9.44	0.728	41757	0.377	29425	0.385	330267
**2**	9	38.83	545	525590	10.39	0.737	40144	0.373	27907	0.376	333060
**2**	10	40.18	551	551160	10.21	0.712	42864	0.387	30695	0.399	334027
**2**	11	41.29	625	557810	9.45	0.731	44930	0.376	32425	0.386	330350
**2**	12	37.69	614	579980	9.62	0.718	46663	0.386	32642	0.388	324548

Table S1. *Cont.*

Id Batch	Id Replicate	S_R	σ_0	η_0	η_{100}	$tan\ \delta$	G_1'	m'	G_1''	m''	AUC
**3**	1	28.28	555	666380	9.40	0.697	63266	0.371	40127	0.347	358830
**3**	2	30.60	555	568810	9.30	0.679	56462	0.368	37441	0.354	312198
**3**	3	28.50	615	573030	9.60	0.670	60576	0.366	39666	0.357	309027
**3**	4	31.35	570	784310	9.20	0.665	56605	0.375	38396	0.364	329339
**3**	5	30.18	557	592620	9.30	0.681	55698	0.364	37578	0.357	317912
**3**	6	28.17	514	729850	9.80	0.691	52792	0.362	35112	0.354	299676
**3**	7	29.15	601	681870	8.70	0.666	55461	0.359	38600	0.371	315850
**3**	8	29.52	564	657560	9.20	0.683	58345	0.362	38274	0.349	338096
**3**	9	28.91	524	703110	8.70	0.693	63830	0.367	42659	0.359	323709
**3**	10	29.15	610	680950	9.10	0.701	58515	0.368	37149	0.347	318245
**3**	11	29.73	578	591990	8.80	0.706	62104	0.363	39793	0.348	332073
**3**	12	30.01	547	645580	8.90	0.673	55899	0.361	36216	0.351	336643
**4**	1	27.31	464	559370	10.80	0.684	51203	0.362	34183	0.353	336761
**4**	2	29.83	369	594390	10.50	0.694	50946	0.365	33480	0.351	350132
**4**	3	28.05	441	557160	10.70	0.689	46430	0.366	31335	0.36	351894
**4**	4	27.67	398	546600	10.80	0.680	52069	0.372	33950	0.353	348860
**4**	5	27.25	390	587910	11.10	0.683	41137	0.37	28258	0.368	362384
**4**	6	26.47	403	558380	11.40	0.707	41079	0.357	27376	0.345	355492
**4**	7	32.18	445	593020	9.20	0.699	45334	0.363	30485	0.365	352674
**4**	8	31.95	404	549970	9.23	0.705	45942	0.371	30449	0.365	339765
**4**	9	31.70	398	585680	9.37	0.694	48813	0.372	32695	0.364	345901
**4**	10	31.71	399	601150	9.13	0.708	44034	0.382	30089	0.38	335165
**4**	11	30.94	402	594720	9.15	0.700	39846	0.371	26763	0.361	338616
**4**	12	31.26	403	557420	8.90	0.698	44010	0.368	29414	0.364	340697
**5**	1	28.03	514	601980	10.09	0.688	56027	0.352	38709	0.355	338366
**5**	2	26.96	555	601660	10.32	0.695	54921	0.379	36293	0.361	338042
**5**	3	27.09	584	595860	9.80	0.685	61309	0.369	41659	0.372	307937
**5**	4	25.06	525	565910	9.96	0.685	58235	0.368	39670	0.364	326831
**5**	5	32.13	537	593330	9.83	0.683	51497	0.367	35288	0.367	336800
**5**	6	28.50	544	568850	9.98	0.704	59227	0.38	39859	0.364	334822
**5**	7	34.26	502	741600	10.20	0.691	64754	0.376	43624	0.364	340707
**5**	8	32.54	545	667510	9.90	0.692	55627	0.35	37803	0.31	330517
**5**	9	34.04	565	675450	9.72	0.702	65003	0.366	43283	0.356	320896
**5**	10	35.90	547	712980	9.19	0.692	62557	0.371	43213	0.374	328907
**5**	11	44.15	554	622860	9.99	0.681	59564	0.371	39078	0.36	333433
**5**	12	33.18	565	665950	9.82	0.695	60235	0.361	39406	0.35	315693
**6**	1	30.04	441	640110	10.47	0.727	43810	0.371	30704	0.38	350372
**6**	2	31.29	514	638680	10.32	0.698	47596	0.374	32972	0.374	348502
**6**	3	31.57	458	585650	10.14	0.707	47665	0.379	32527	0.377	336643
**6**	4	33.04	469	542150	9.94	0.726	49500	0.369	33425	0.385	351055
**6**	5	31.32	500	530220	9.73	0.713	54661	0.367	33320	0.369	353293
**6**	6	31.61	489	541410	9.98	0.727	48100	0.371	37127	0.374	337585
**6**	7	44.37	498	640720	10.09	0.712	44566	0.379	30486	0.378	354648
**6**	8	32.04	457	542990	10.02	0.705	47337	0.357	32852	0.371	351379
**6**	9	31.59	500	621160	9.53	0.707	46528	0.375	31815	0.375	351899
**6**	10	44.56	476	688780	9.46	0.694	49733	0.362	33636	0.359	353489
**6**	11	36.67	444	599560	9.58	0.711	53170	0.379	35900	0.369	354049
**6**	12	41.92	510	520290	9.86	0.712	50375	0.363	34744	0.366	349719
**7**	1	27.09	517	839980	8.59	0.662	55026	0.357	42111	0.341	356484
**7**	2	25.18	560	779980	8.05	0.665	53687	0.334	34631	0.340	347010
**7**	3	28.45	578	712780	8.40	0.655	61096	0.360	35784	0.343	352714
**7**	4	26.02	545	717580	8.41	0.671	57763	0.353	38367	0.353	365147
**7**	5	25.99	546	739780	8.41	0.675	59618	0.355	37601	0.352	347010
**7**	6	25.78	540	644530	8.60	0.654	62899	0.335	37530	0.342	354977

Table S1. *Cont.*

Id Batch	Id Replicate	S_R	σ_0	η_0	η_{100}	$tan\ \delta$	G'_1	m'	G''_1	m''	AUC
**7**	7	28.76	555	755085	8.35	0.674	57388	0.347	36956	0.340	365663
**7**	8	28.24	548	737850	8.45	0.651	66661	0.358	43183	0.350	354471
**7**	9	26.83	565	744070	8.11	0.670	58266	0.361	36958	0.345	356160
**7**	10	30.11	571	702410	8.27	0.658	52788	0.355	33678	0.351	358005
**7**	11	26.40	532	745450	8.27	0.657	52738	0.358	33404	0.342	362688
**7**	12	28.35	512	820940	8.26	0.654	58888	0.358	39453	0.364	357259
**8**	1	31.55	527	725070	10.26	0.725	51834	0.368	35138	0.366	346504
**8**	2	34.25	489	602700	10.75	0.729	51640	0.372	35496	0.375	379991
**8**	3	36.60	515	651890	10.56	0.721	53050	0.38	36615	0.383	368372
**8**	4	36.64	502	603320	10.39	0.717	60185	0.379	34367	0.381	374145
**8**	5	36.37	498	640210	10.20	0.712	53122	0.385	41454	0.385	360440
**8**	6	37.29	515	611540	10.35	0.711	60185	0.379	37234	0.391	361466
**8**	7	37.89	485	703600	9.75	0.710	51267	0.383	34832	0.382	367268
**8**	8	38.16	510	659460	10.51	0.711	46415	0.369	32514	0.377	355963
**8**	9	38.40	520	690680	10.27	0.728	55331	0.38	37644	0.374	357136
**8**	10	38.88	490	635190	10.16	0.721	57526	0.376	39720	0.382	357652
**8**	11	38.18	504	641910	9.58	0.715	48860	0.376	31845	0.358	368696
**8**	12	37.74	515	631690	9.87	0.719	50549	0.37	35413	0.381	358786
**9**	1	34.88	430	488890	9.84	0.699	49940	0.369	33428	0.364	364445
**9**	2	33.89	476	674650	9.06	0.709	52849	0.376	35678	0.362	358854
**9**	3	33.99	500	643240	8.70	0.707	60845	0.376	40396	0.361	342195
**9**	4	33.13	485	579900	9.35	0.710	51443	0.381	35174	0.38	355143
**9**	5	35.25	437	577110	9.74	0.705	53090	0.369	36146	0.365	340501
**9**	6	35.94	459	548750	9.66	0.703	48207	0.375	32457	0.363	338567
**9**	7	36.17	455	618640	9.61	0.707	54285	0.368	35691	0.352	350171
**9**	8	34.87	501	651940	9.75	0.712	52379	0.368	35047	0.36	352714
**9**	9	35.26	436	630620	9.51	0.698	56733	0.375	37206	0.354	348070
**9**	10	35.17	448	606220	9.68	0.694	48380	0.371	33156	0.365	332721
**9**	11	35.69	464	604360	9.63	0.707	51249	0.381	34791	0.368	352714
**9**	12	36.29	476	668510	9.79	0.705	49231	0.364	33491	0.362	348365
**10**	1	40.08	584	768370	9.60	0.697	63833	0.367	43117	0.357	292922
**10**	2	40.45	564	718070	9.40	0.696	66955	0.386	43289	0.36	328043
**10**	3	40.15	559	718980	9.70	0.700	65966	0.375	42766	0.344	350392
**10**	4	39.62	560	726270	9.90	0.694	70941	0.377	46480	0.352	336785
**10**	5	40.19	579	724360	9.30	0.679	60911	0.373	40928	0.363	330959
**10**	6	39.17	580	738550	9.50	0.690	67503	0.363	48626	0.376	326600
**10**	7	40.59	564	693890	9.60	0.681	60021	0.369	41153	0.362	341689
**10**	8	39.74	564	782160	9.70	0.692	62167	0.367	40556	0.350	342219
**10**	9	40.15	558	693920	9.10	0.708	65875	0.374	43604	0.360	332829
**10**	10	40.11	559	621020	9.40	0.678	66925	0.378	43265	0.360	319556
**10**	11	39.62	571	663060	9.20	0.674	62487	0.371	42478	0.370	343608
**10**	12	39.52	563	615410	9.30	0.679	76270	0.364	49457	0.343	329118
**Mean**		33.80	519	630067	9.63	0.700	53255	0.369	35829	0.365	342224
**SD**		4.82	57	74229	0.67	0.020	7741	0.010	4723	0.015	15438
**CV (%)**		14.3	11.0	11.8	7.0	2.9	14.6	2.6	13.2	4.1	4.5

S_R, relative thixotropic area; σ_0, yield stress; η_0, zero-shear viscosity; η_{100}, viscosity at 100 s^{-1}; *tan δ*, loss tangent at 1 Hz; G'_1, calculated elastic modulus; G''_1, calculated viscous modulus; *m′* and m'' are the parameters obtained when fitting *G′* and *G′′*, respectively, *versus* frequency; *AUC*, area under the weight *versus* surface curve (spreadability).
